# *C’est vraiment compliqué*: a case study on the delivery of maternal and child health and nutrition interventions in the conflict-affected regions of Mali

**DOI:** 10.1186/s13031-020-0253-6

**Published:** 2020-05-27

**Authors:** Anushka Ataullahjan, Michelle F. Gaffey, Moctar Tounkara, Samba Diarra, Seydou Doumbia, Zulfiqar A. Bhutta, Diego G. Bassani

**Affiliations:** 1 Centre for Global Child Health, the Hospital for Sick Children, Toronto, Canada; 2 Faculty of Medicine and Odontostomatology, University of Sciences, Techniques and Technology of Bamako, Bamako, Mali; 3 University Clinical Research Center, Bamako, Mali; 4 Center of Excellence in Women and Child Health, Aga Khan University, Karachi, Pakistan

**Keywords:** Mali, Conflict, Humanitarian emergency, Children, Newborn, Adolescent, Women, Health

## Abstract

**Abstract:**

**Background:**

Mali is currently in the midst of ongoing conflicts which involve jihadist groups, rebels, and the state. This conflict has primarily centered in the North of the country. Humanitarian actors delivering services in these geographies must navigate the complex environment created by conflict. This study aimed to understand how humanitarian actors make decisions around health service delivery within this context.

**Methods:**

The current case-study utilized a mixed methods approach and focused on Mopti, Mali’s fifth administrative region and fourth largest in population. Latent content analysis was used to analyze interview transcripts guided by our research objectives and new concepts as they emerged. Indicators of coverage of health interventions in the area of maternal and child health and nutrition were compiled using Mali’s National Evaluation Platform and are presented for the conflict and non-conflict regions. Development assistance estimates for Mali by year were obtained from the Developmental Assistance for Health Database compiled by the Institute for Health Metrics and Evaluation. Administrative data was compiled from the annual reports of Mali’s Système Local d’Information Sanitaire (SLIS), Demographic and Health Surveys (DHS) and Multiple Indicator Cluster Surveys (MICS).

**Results:**

Our data suggests that the reaction of the funding mechanisms to the conflict in Mali was a major barrier to timely delivery of health services to populations in need and the nature of the conflict is likely a key modifier of such reaction patterns. Concerns have been raised about the disconnect between the very high administrative capacity of large NGOs that control the work, and the consequent burden it puts on local NGOs. Population displacement and inaccurate estimates of needs made it difficult for organizations to plan program services. Moreover, actors delivering services to populations in need had to navigate an unpredictable context and numerous security threats.

**Conclusions:**

Our study highlights the need for a more flexible funding and management mechanism that can better respond to concerns and issues arising at a local level. As the conflict in Mali continues to worsen, there is an urgent need to improve service delivery to conflict-affected populations.

## Background

Since early 2012, the lives of many in Mali, especially those living in the country’s northern regions, have been disrupted by a complex and evolving conflict that has led to high insecurity, unpredictable waves of civil unrest, and ongoing political instability [1].

The collapse of Libya in 2011 dispersed vast numbers of armed Jihadist groups and their weapons to other countries in the region, including Mali. This influx further exacerbated existing ethnic tensions in the country, with violent conflict eventually erupting between local ethnic minorities and the Malian government in Northern Mali in January 2012 [2]. This violence was the culmination of historical tensions between the government and the Mouvement National pour la Libération de l’Azawad (MNLA), a group comprised of ethnic minorities of northern Mali, fighters returning from Libya, and deserting soldiers from the Malian army [3].

The origins of these tensions are best understood in light of Mali’s history, varied geography and complex ethnic fabric that have combined to produce a rich culture but also large developmental inequalities between regions, perpetuating historical internal divisions [3]. Although internal tensions have existed for centuries, violence escalated when the MNLA joined forces with the Tuareg-led Jihadist group Ansar Dine, the Algerian-based group Al-Qaeda in the Islamic Maghreb (AQIM), and its west Africa offshoot, the Movement for Oneness and Jihad in West Africa (MOJWA) against the Malian government in pursuit of greater autonomy and independence for northern Mali through the creation of the Azawad state. These actors exploited pockets of population dissatisfaction with the state in northern Mali and were able to garner initial support against the Malian government [4].

In March 2012, the government’s inability to effectively address the violence and the evolving occupation of Northern Mali by Jihadists resulted in a *coup d’état* that exacerbated the instability and led the national army to withdraw from the northern regions, resulting in the rebel domination of most large cities in all four northern regions [5]. The MNLA proclaimed the independence of northern Mali shortly after, but by mid-2012 a wider Jihadist-nationalist conflict emerged between the population, the MNLA, AQIM/MOJWA, and Ansar Dine [2]. Violence escalated in the region during the second half of 2012, and in January 2013, by request of the Malian government and the Economic Community of West African States (ECOWAS), French military intervention began, soon resulting in the government reasserting control of the northern region and in a short-lived cease-fire and realignment of the MNLA with the Malian government in the fight against the Jihadists.

The de-escalation of the conflict by the French military intervention was followed by the deployment, in early July 2013, of the United Nations Multidimensional Integrated Stabilization Mission in Mali (MINUSMA), a peacekeeping mission with the goal of stabilizing the region [6]. MNLA and the Malian government ended their ceasefire in September 2013 and clashes between the Jihadist groups and the state continued. Another ceasefire and subsequent peace agreement was signed on April 2015, but expectations that the crisis was nearing its end have not been realized. The agreement does not formally apply to central Mali (Mopti region), where the ethnic and religious nature of the conflict, led by radical groups, has continued in contrast with the ending of the rebellion in the north [6].

In a country still rife with ongoing insecurity, the transition of the humanitarian response into developmental assistance continues to face multiple barriers [7] impacting international development assistance operations in Mali. In March 2012, immediately after the *coup d’état*, many large international donors halted their official development assistance to the country [5]. Those that did not suspend activities shifted the management and delivery of their operations from government to non-governmental organizations (NGOs). The move away from supporting government-led programs seriously compromised the functionality of the public health sector, which decreased further when medical officers and regional health directors left the region due to the growing insecurity and, more specifically, the anti-government nature of the tensions. The government withdrawal from the region and the transition of donor funding from increasingly centralized developmental assistance to decentralized humanitarian response in northern Mali prompted the activation by the United Nations Office for the Coordination of Humanitarian Affairs (OCHA) of the cluster system in 2012, to ensure coordination of the provision of care to the conflict-affected populations [5].

Delivering programs and services to Mali’s 19.3 million habitants is challenging for the more than 150 organizations operating in multiple sectors (protection, education, food security, water, sanitation, hygiene, coordination, health, nutrition and shelter) [6]. Coordination of such a large number of actors is complex, the evolving and unpredictable nature of the conflict makes planning difficult, and both financial and human resources are limited. In 2017, staff from over 85% of these organizations were targeted in carjacks or were victims of theft and/or physical aggression during the course of their work [6].

Identifying strategies to overcome the barriers to delivering services to conflict-affected populations is essential in ensuring their health needs are met. As described above, the ongoing conflict in northern Mali is complex, as are its implications for service provision. This objectives of this study were to better understand the influence of the conflict on the planning, coordination and delivery of maternal and child health and nutrition interventions in northern Mali; to identify the strategies used to overcome obstacles to health intervention delivery during the conflict; and to inform best practices in the future delivery of health and nutrition services in Mali and other conflict settings.

## Methods

This study is part of a series of case-studies coordinated by the BRANCH Consortium with the objective of understanding the delivery of reproductive, maternal, newborn, child and adolescent health and nutrition programs in ten conflict-affected countries: Afghanistan, Colombia, Democratic Republic of Congo, Mali, Nigeria, Pakistan, Somalia, South Sudan, Syria, and Yemen. The current case-study used a mixed-methods approach [8] and focused on Mopti, one of Mali’s administrative regions and fourth largest in population.

### Document review

A desk review of the English and French language literature including peer-reviewed publications, program reports, and cluster meeting minutes was conducted to identify relevant documents, also including searches of relevant databases with publications and information targeting and/or generated by humanitarian organizations. An exercise to map humanitarian activity in the country since the beginning of the conflict was also conducted, using information available in program reports and websites, ‘Who does What Where’ (3 W) maps [9], and OCHA cluster meeting minutes.

### Quantitative data

Indicators of coverage of health interventions in the area of maternal and child health and nutrition were compiled using Mali’s National Evaluation Platform and are presented for the conflict (Gao, Kidal, Mopti, Segou, Tombouctou) and non-conflict (Bamako, Kayes, Koulikoro and Sikasso) regions of Mali separately [10, 11]. Development assistance estimates for Mali by year were obtained from the Developmental Assistance for Health Database compiled by the Institute for Health Metrics and Evaluation [12]. Administrative data were compiled from the annual reports of Mali’s Système Local d’Information Sanitaire (SLIS) from 2000 to 2016 [13] as well as Demographic and Health Surveys (DHS) [14–19] and Multiple Indicator Cluster Surveys (MICS) [20, 21].

### Qualitative data

Qualitative interviews were conducted between August and September of 2018. Three male data collectors, with previous experience in conducting qualitative interviews, were hired to conduct the interviews and were trained on basic interview techniques and on the objectives of the study. The data collectors had varying backgrounds including training in anthropology, social sciences and medicine.

Data were collected through semi-structured interviews lasting between one and 2 h. Participant observation was used on one occasion, when the study team attended a health cluster meeting in Bamako and focused on the activities undertaken during the meeting, as well as the interactions between different actors.

A total of 50 respondents, representing 50 different organizations were interviewed. Individuals were selected using purposive sampling targeting organizations that worked in any area of RMNCAH&N (Reproductive, Maternal, Newborn, Child, Adolescent Health and Nutrition) in the Mopti region. These included UN agencies, inter-governmental organizations, government, and international and local non-governmental organizations involved in the planning, coordination and delivery of maternal and child health and nutrition interventions in the northern regions of Mali. Target organizations were identified during the document review phase and were contacted by the study team (by telephone and/or e-mail) to briefly present the study objectives. We then identified individuals within these organizations who had extensive experience in delivering services to conflict-affected populations and could speak to our study objectives and subsequently scheduled interviews. The data collectors conducted interviews in locations that chosen by the respondents, and the majority of the interviews occurred at the respondents’ own offices.

A semi-structured interview guide included questions focused on the organizations’ activities planned and/or implemented during the period between 2013 and 2018. Areas of investigation included understanding the role of considerations such as the availability of finances, health workers, and commodities on the decision making of humanitarian organizations embedded within a specific sociocultural, and security context. The findings presented within this manuscript are based on the 25 most informative interviews (Table 1).

**Table 1:** Description of respondents

Respondents	Number
Government Representatives	5
UN Agencies and Multi-Country Commissions	5
Local NGOs	2
International NGOs	13

Interviews were conducted in French, and audio recorded where permission was granted. One respondent refused to have their interview recorded. Interviews were transcribed in French and excerpts were translated to English for inclusion in this manuscript.

Latent content analysis was used to analyze the data. Transcripts were manually coded in ATLAS.ti™. Local investigators conducted the preliminary coding for the analysis, with further coding and analysis conducted by the team of investigators based in Canada. Member checking was used to increase to the rigor of our analysis. Preliminary findings were shared with representatives from humanitarian organizations and regional ministry of health officials during a dissemination meeting held in Bamako in December 2018. The discussions that occurred during this meeting helped to further guide and refine our analyses and interpretation of results.

Our final analysis sought to explain the major conflict-related factors affecting planning, coordination and delivery of maternal and child health and nutrition interventions in northern Mali by integrating the findings from: (i) the qualitative interviews, in which members of the organizations describe their perspectives; (ii) contextual information about the conflict based on the document review; (iii) a descriptive quantitative analysis of several data sources containing information on the flow of financial resources from international donors for developmental assistance in health into the country, number and timing of conflict events and battle related deaths and (iv) the coverage of interventions in the conflict and non-conflict affected regions of the country over time.

Ethics approval was received from both the Research Ethics Board from the Hospital for Sick Children and the Faculty of Medicine and Odontostomatology at the University of Sciences, Techniques and Technology of Bamako, Mali.

## Results

**Fig. 1:**
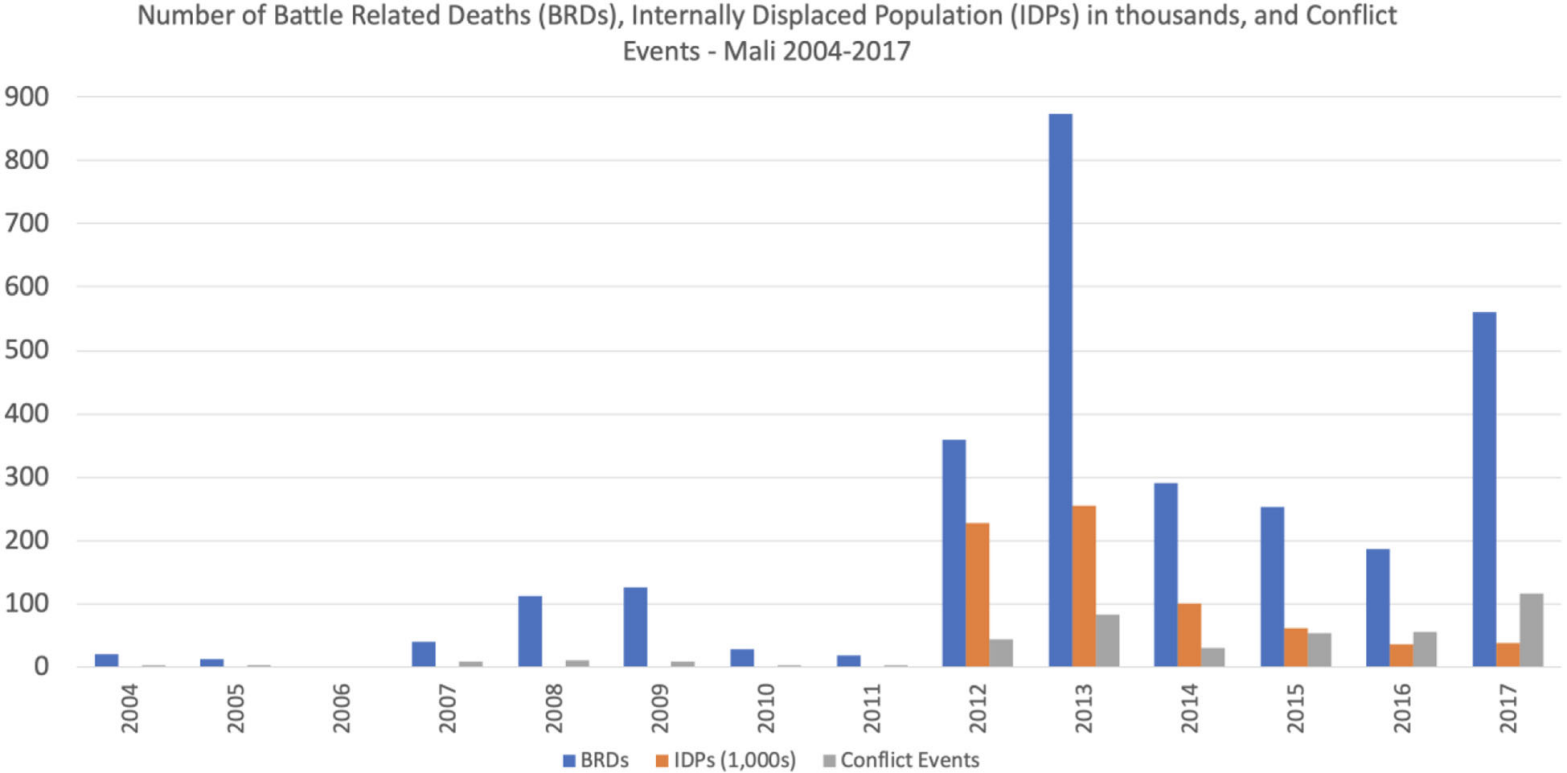
Number of Battle Related Deaths (BRDs), Internally Displaced Population (IDPs) in thousands, and Conflict Events - Mali 2004–2017

The estimated number of battle related deaths (BRDs) in Mali rose sharply in 2012 and to a peak of nearly 900 in 2013, with a further spike to more than 500 in 2017 (Fig. 1). The number of internally displaced people (IDP) was estimated to be 227,000 in 2012, and increased to more than 250,000 in 2013. Even though IDPs started to return to their areas of origin after 2013, the tensions have not dissipated and the UN still classifies its Malian peacekeeping mission as the deadliest of this decade [22].

**Fig. 2:**
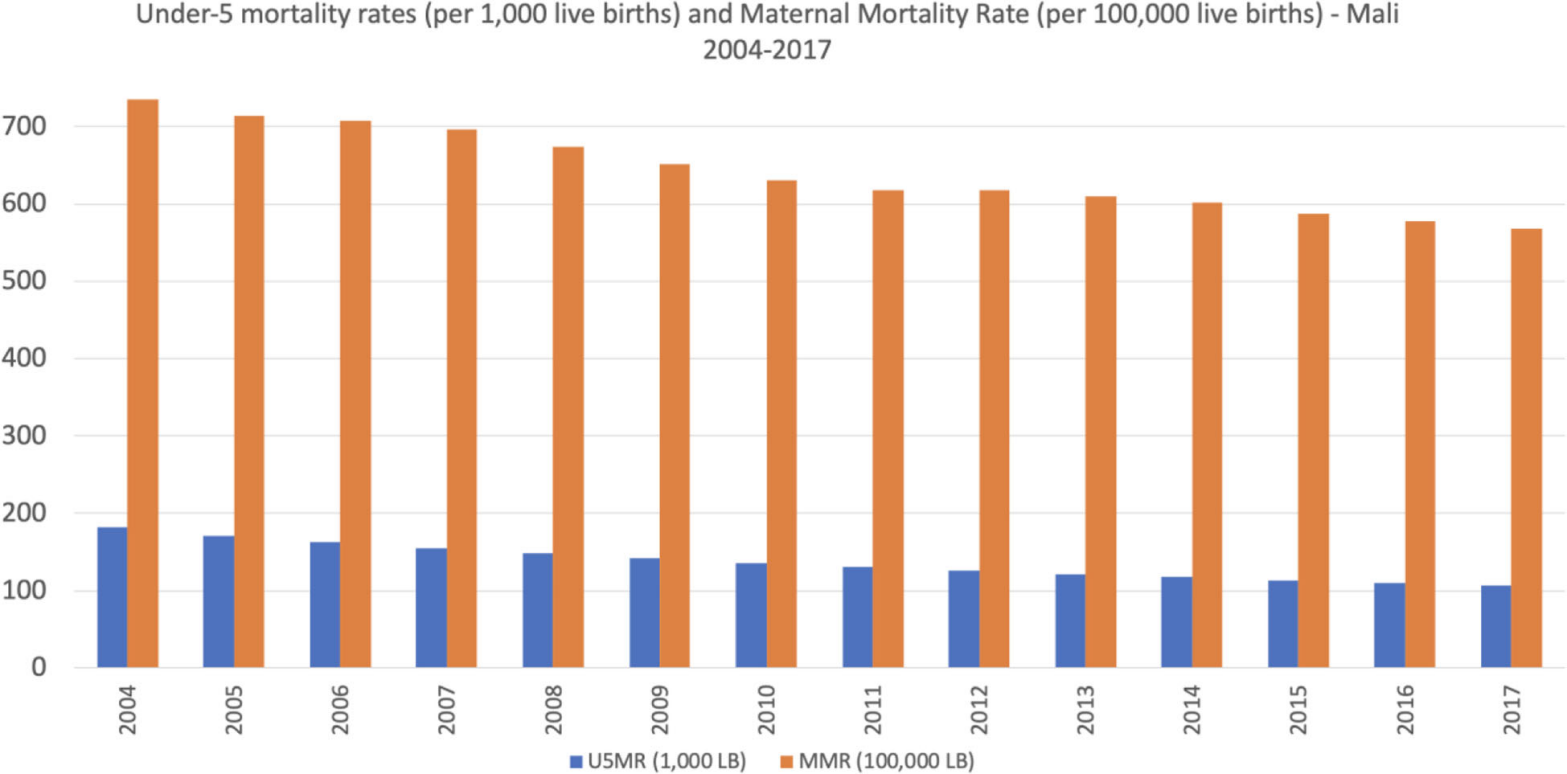
Under-5 mortality rates (per 1000 live births) and Maternal Mortality Rate (per 100,000 live births) - Mali 2004–2017

Mali currently has one of the world’s highest under-five mortality rates, estimated to be 106/1000 live births in 2017, and very high maternal mortality rates, estimated to be 567/100,000 live births in 2017 (Fig. 2) [23].

These extremely high mortality rates are due in part to longstanding difficulties in delivering and accessing health services in Mali, especially for women and children; a situation that has been aggravated by the conflict. The most recent national estimates, from the 2018 DHS, indicate that only 67% of women deliver in health facilities, and only 67% of deliveries are attended by a health provider [24]. Moreover, this national survey also demonstrated that 27% of children under 5 years of age were stunted or suffered from chronic malnutrition [24]. These statistics indicate the need for increasing focused services for women and children. Although there have been improvements in many maternal health indicators in Mali, regional variations continue to persist [25].

The qualitative interviews revealed that while conflict is perceived as a pervasive contextual factor that impacts maternal and child health and the health needs of the population, it rarely figured prominently as an explicit driver of program planning. The recency of the conflict relative to funding and program implementation cycles may account in part for the apparent downplay of its role. However, this can also be interpreted as a consequence of the fragmentation of the program delivery structures; at present, international donors fund international agencies that support their offices in Mali, which, in turn, may partner with local organizations for program delivery in the conflict-affected regions. In the conflict areas, government withdrawal due to direct targeted attacks has resulted in a continuing reduction in the number of functional health facilities: while 88% of the government health facilities were functional in 2012, the number of functional facilities in 2017 was 83% and decreased to 78% in 2019 [26–28], mostly due to insecurity and the ongoing targeted attacks on health facilities and personnel [29]. The number of skilled health workers has reduced by 31% and in addition, 80% of the remaining health workers are paid by through international developmental assistance funds [6].

Although the security situation is often recognized as an important barrier for program delivery, and an important driver of operational costs, it did not emerge as a factor directly connected to the early planning and prioritization of programs and interventions. Instead, it was clear, from multiple interviews, that the most prominent driver of program prioritization and planning were donor demands. On that front, the conflict was perceived as a catalyst for stricter donor stipulations on fund allocation, limiting the organizations’ ability to address changes in needs that result from the conflict. Respondents, and their organizations, struggled to reconcile their recognition of the emerging population needs with their inability to respond with appropriate programming whenever these needs fell outside of the scope of the funding available. Respondents did not differentiate between development and humanitarian donors when describing funding stipulations. The following sections detail our findings describing how the conflict affected internationally funded health programs in Mali.

### Funding: constraint and inflexibility

Donor inflexibility and the consequent difficulty in diverting funds to activities for which they were not earmarked was described in several interviews, and respondents suggested these characteristics were exacerbated by the conflict. According to one respondent, the presence of conflict increased donor’s concerns that funds could be misappropriated and/or used to support terrorist activities. Despite hypothesized justifications for donor inflexibility, funding restrictions limited the organizations’ ability to comprehensively address the health needs of the population and even as the conflict unfolded, funded programs were rarely supplemented to address the changing health needs or to accommodate increases in the number of program beneficiaries due to population displacement. Instead, they struggled to serve larger populations within the allocated resources.

The amount of funding available for health programs was impacted by the conflict, despite initial surges in the overall amount of resources entering the country. Respondents noted during interviews that when the conflict began there was an immediate influx of funding, however, it has not been sustained despite the continuation of the crisis.

*As I said, in 2013 when [our organization] was hit by the crisis, donors immediately mobilized resources to help [us] intervene. Consequently, we had a lot of resources. Since the situation started improving, compared to the years of 2013 and 2014, resources have been decreasing. (UN agency employee).*

**Fig. 3:**
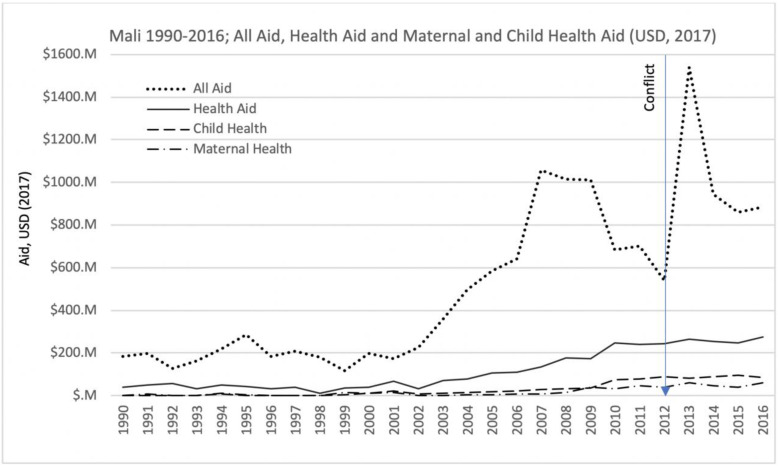
Mali 1990–2016 All Aid, Health Aid and Maternal and Child Health Aid - Developmental Assistance for Health Database [12]

The analysis presented in Fig. 3 confirms this observation. In fact, during 2012, there appears to have been a considerable decrease in funding available. As indicated by our respondents, donor delays in releasing funds in response to or as a consequence of the conflict, and even complete interruptions in funding, are evident from an analysis of the Developmental Assistance for Health Database, [12] where a dramatic reduction in financial assistance flowing into Mali during 2012 is confirmed (Fig. 3), with the increase in resources being observed only in 2013, at least 11 months after the conflict started.

The dramatic initial reduction in the total amount of international assistance funding for Mali does not seem to have been as prominent in health program funding, however. That being said, the small but steady increase in health financing for the country that started at least 5 years before the conflict has stalled since 2012. The lack of a continued increase in the amount of international assistance funding allocated to health is important when one considers the increased health needs due to conflict. There may also be important differences in the amount of resources allocated to the conflict and non-conflict areas, but it is not possible to investigate this as the data are only available in aggregated form for the whole country,

Funding restrictions and/or limitations meant that organizations needed to prioritize selected services even though they felt comprehensive services were needed in the conflict areas. A reduction in the geographic scope of the programs was a common programmatic response to the barriers imposed by inflexible funding structures and the weaning amount of resources available.

Our work is determined by the funding we have. I take the example of the Mopti project that was implemented in 2013 and early 2014. We wanted to expand it across the district of Mopti and the Mopti region. We did not get any funding so it is certain that the indicators [used to track the services we were providing] in Mopti decreased, because we did not have funding. (International NGO employee)

Program planning is highly dependent on donor decisions and constraints imposed by earmarking resources. The geographical scope could also be determined by the funder, by the security situation and by the available financial resources. Respondents suggested that organizations mostly make use of available data and national directives to decide on the details of programs and services to deliver and the implementation strategies, but not on the types of programs (e.g., nutrition, immunizations, obstetric care) or geographical areas of focus as these were pre-determined by funders.

In 2016, of the 274 million US dollars in international assistance funding available for health (30% of all international assistance for the country), only 30% (83.7 M) was allocated to child health programs and about 20% (59.5 M) to maternal health, and there has been virtually no change in the amount of international funds available for maternal and child health programs in the country since the conflict started in early 2012.

**Fig. 4:**
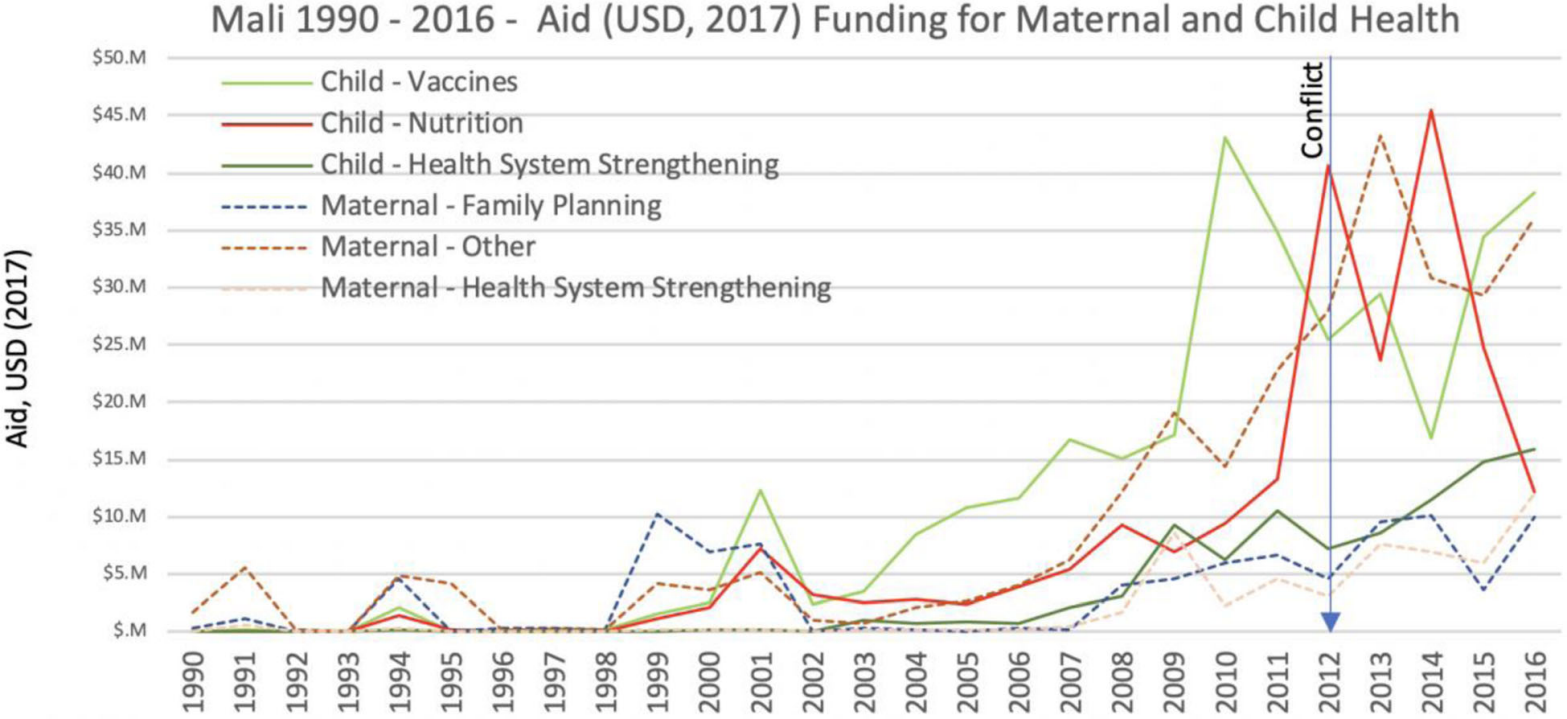
Mali 1990–2016-Aid Funding for Maternal and Child Health - Developmental Assistance for Health Database [12]

The disaggregation of funding allocation by programmatic area reveals that resources were immediately higher at least in the area of child nutrition and there have been more steady increases in the area of health system strengthening, though the amount of resources was still limited to less than 16 M dollars in 2016. Maternal health funding from international donors has also been unstable since the conflict began (Fig. 4).

**Fig. 5:**
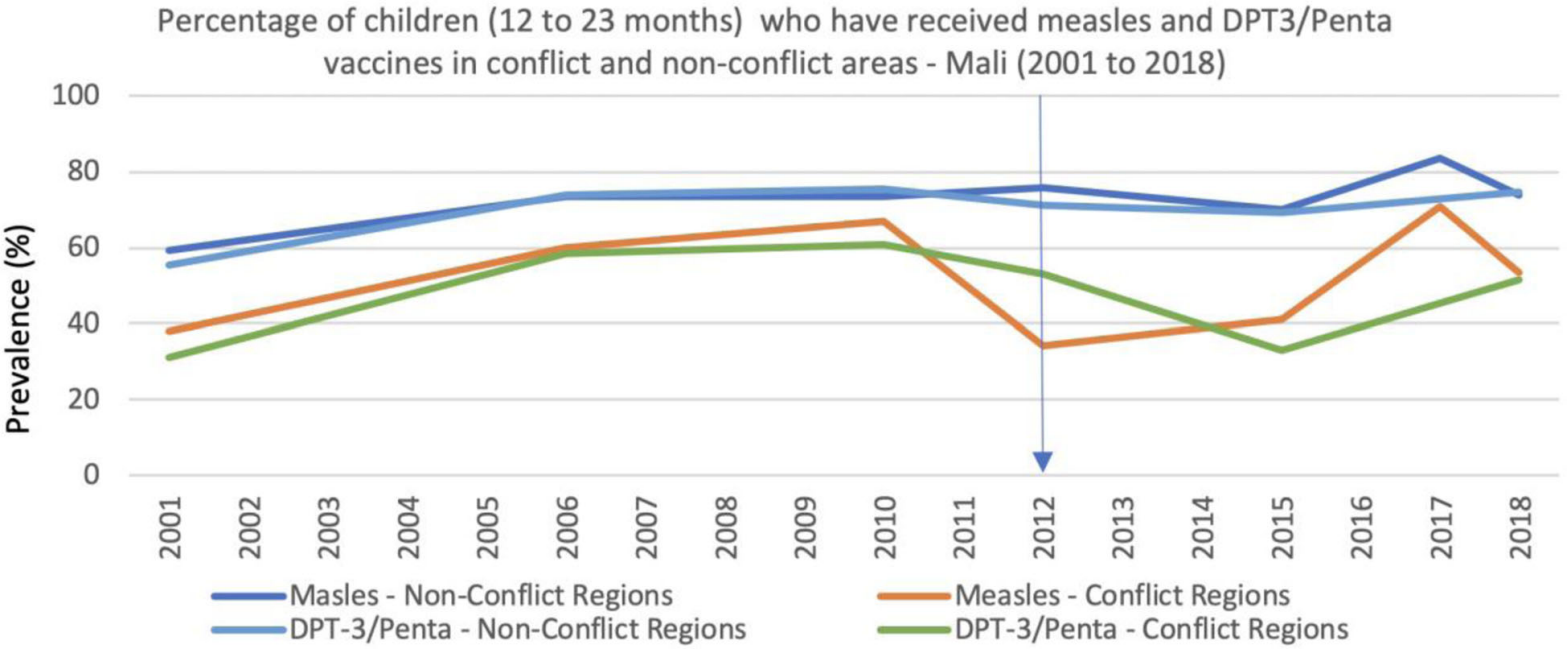
Percentage of children (12 to 23 months) who have received measles and DPT3/Penta vaccines in conflict and non-conflict areas - Mali (2001 to 2018)

Dramatic decreases in the funding for childhood immunization between 2010 and 2012 and even further declines in 2014 are evident from the analysis of the Developmental Assistance for Health Database [12] (Fig. 4). These declines likely contributed to the important reduction in measles vaccination coverage among children 12 to 23 months in the conflict regions in 2012 (Fig. 5). Coverage of measles vaccine in the region only increased again in 2015 and then further in 2017. International funding for childhood immunizations also increased in 2015 and 2016, in response to the serious measles outbreaks that occurred in northern Mali in all years between 2012 and 2016 (260 cases per year on average) and again in 2018/2019 (950 cases in 14 months). This is the highest number of cases of measles in Mali since the 2009/2010 outbreak.

In contrast to the response to the measles outbreaks described above, coverage of DPT3 vaccine has declined in conflict regions. In 2015 coverage reached only 30%, one of the lowest levels since 2001 (Fig. 5). These quantitative findings were supported by our qualitative data where several respondents cited financial constraints as a barrier to providing immunizations. It is, however, important to note that financial constraints are one of many factors affecting immunization rates, and the role of availability of commodities, access, and security constraints have also been identified by respondents as strong barriers to service provision, as discussed further below.

It is clear that delays in funding after the conflict began were compounded by funding reductions that limited program availability, reduced their duration and limited the quality of the services delivered. Changes in funding and delays also forced organizations to enact unplanned adjustments to the programs, which inevitably increased operational costs both during implementation but also while the organizations struggled to identify new delivery strategies. For example, access to certain geographies changes seasonally and timing the delivery of programs accordingly is a strategy to reduce program delivery costs. Interventions that were planned to be delivered to areas accessible by car during the dry season have much higher costs if they are ultimately delivered during the rainy season as a consequence of funding delays. In this specific situation, workers had to use boats to access the areas due to floods; because motorized boats were far more expensive than cars, the program was entirely delivered using row boats instead, increasing delivery time significantly.

Imagine if you have to work in the Delta this August, September, October. There is water everywhere so you have to use a motorized boat which requires a liter of fuel to cover one kilometer. So instead of using 15 liters of fuel to drive 100 kilometers with a car, you have to use 100 litres to go the same distance with a boat. But how are you going to wait for the dry season? This is often our issue with partners, funds do not come in time. Yet it is only between January and June that these areas are accessible by car. If funding comes late, the areas are no longer accessible, then we have to use the phone, the radio or other means of communication. (International NGO employee)

### Programming: scepticism and displacement

Despite the strong influence of funding, there was still important variability in the resources used to inform the selection of specific interventions and services, delivery strategies and geographic scope. Some organizations relied primarily on government reports, some referred to research evidence, and others based their decisions on previous experience. Respondents also highlighted the use of monitoring and evaluation strategies to inform programming and improve services. Overwhelmingly, respondents maintained that they hoped to deliver services that reflected the community’s needs, despite our respondents’ clear perception that the funders were primarily responsible for determining programming and for the funding inflexibility highlighted earlier. They also demonstrated a clear knowledge of a sound health planning logic that is based on the best available contextual evidence, despite pragmatic realities often not aligning with this sound logic.

When determining priorities, we must look at the problem in terms of the importance of effects on the population, the capacity that the country has to address the problem, the potential the intervention has to make positive changes - we must take all of these things into consideration when trying to determine priorities. (UN agency employee)

Multiple respondents indicated that the actual planning of service delivery strategies, however, was difficult because of a perceived inaccuracy of, or mistrust in, quantitative estimates of needs. This general lack of trust in available data stemmed at least in part from a perceived inability of survey methodologies to accurately capture the needs of a population, with implications for program planning.

For nutrition, we use the SMART nutritional surveys, surveys that are internationally recognized and accepted. So yes; they actually influence [program planning]. For example, last year, the surveys indicated that [a certain number of children were malnourished], after much discussions, and coming to a consensus, we validated the data together. But the number of children who need treatment in Mali had been underestimated, and the resources that the funders put in place [were not sufficient]. They're giving you what you've asked for, but the country [programs], therefore, become wobbly with insufficient resources [to treat all the children in need], because initially there was that [an underestimate of needs]. (International NGO employee)

Migration and internal displacement contributed to the perception that data were unreliable. Population movements during conflict situations often occur rapidly and are highly responsive to evolving events, and from the perspective of some of the interviewees, these movements served as a reason to mistrust data, and were interpreted as another example of inaccurate estimates of population sizes. That being said, regardless of the interpretation of the phenomenon, under-estimates of population sizes resulted in shortages of supplies and commodities.

Often the stocks that we bring are estimated based on the needs of 50 or 100 people but then we realize that the demand is higher, which often results in difficulties. (UN agency employee)

### Population health outcomes: gains and losses

**Fig. 6:**
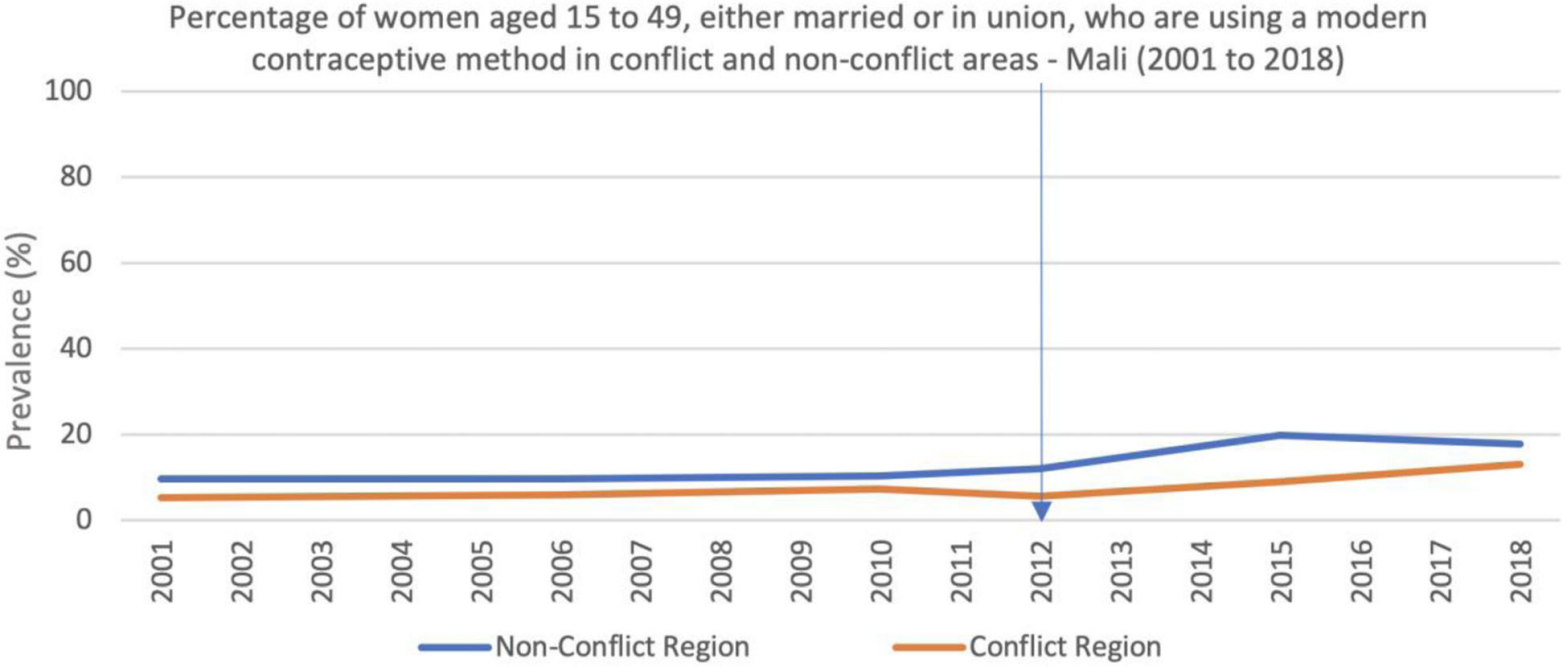
Percentage of women aged 15 to 49, either married or in union, who are using a modern contraceptive method in conflict and non-conflict areas - Mali (2001 to 2018)

Comparative changes in the coverage of health indicators in conflict and non-conflict regions corroborate many of the perceptions of the organizations interviewed. For example, in contrast to the limited but persistent increases in access to modern contraceptives that are observed in the non-conflict regions of Mali since 2010, the conflict regions experienced an immediate reduction as the conflict began and only a very modest increase in the last 6 years (Fig. 6).

**Fig. 7:**
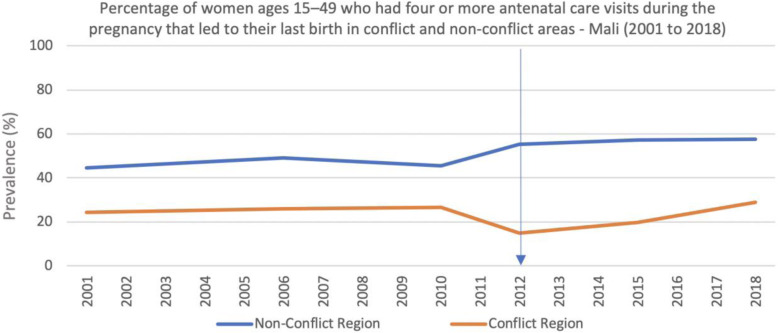
Percentage of women ages 15–49 who had four or more antenatal care visits during the pregnancy that led to their last birth in conflict and non-conflict areas - Mali (2001 to 2018)

The qualitative interviews suggested that respondents believe the conflict had a large impact on family planning services, with many considering it too dangerous to provide family planning services in the current context, fearing that they would become targets of jihadist groups if they delivered these services. This fear did not always translate into the interruption of such services however, as some organizations did continue to provide family planning services. One respondent described how community members would present for family planning services at night in order to circumvent security risks and societal pressures, and organizations would respond accordingly by providing services at these times. Similarly, access to antenatal care increased in the non-conflict regions after 2010, had but decreased in the conflict regions (Fig. 7).

Another complicating factor that coincided with the conflict in Mali, was USAID’s suspension of funding to organizations that include provision of abortion services in any of their programs in any country. This decision had far-reaching implications and contributed to an even more difficult situation that impacted programs and services beyond reproductive health. Some respondents described that they needed to choose between providing abortion services and receiving funding.

With the cuts in the US funding, we were forced to lay off some of our providers and to even reduce the scope of our interventions. When funding suddenly stops, it generates a lot of issues. Even the vehicles that had been purchased for the project were returned to the project [funder]. The motorcycles were returned to the funder. It reduces the resources in terms of personnel and intervention on the ground. (International NGO employee)

**Fig. 8:**
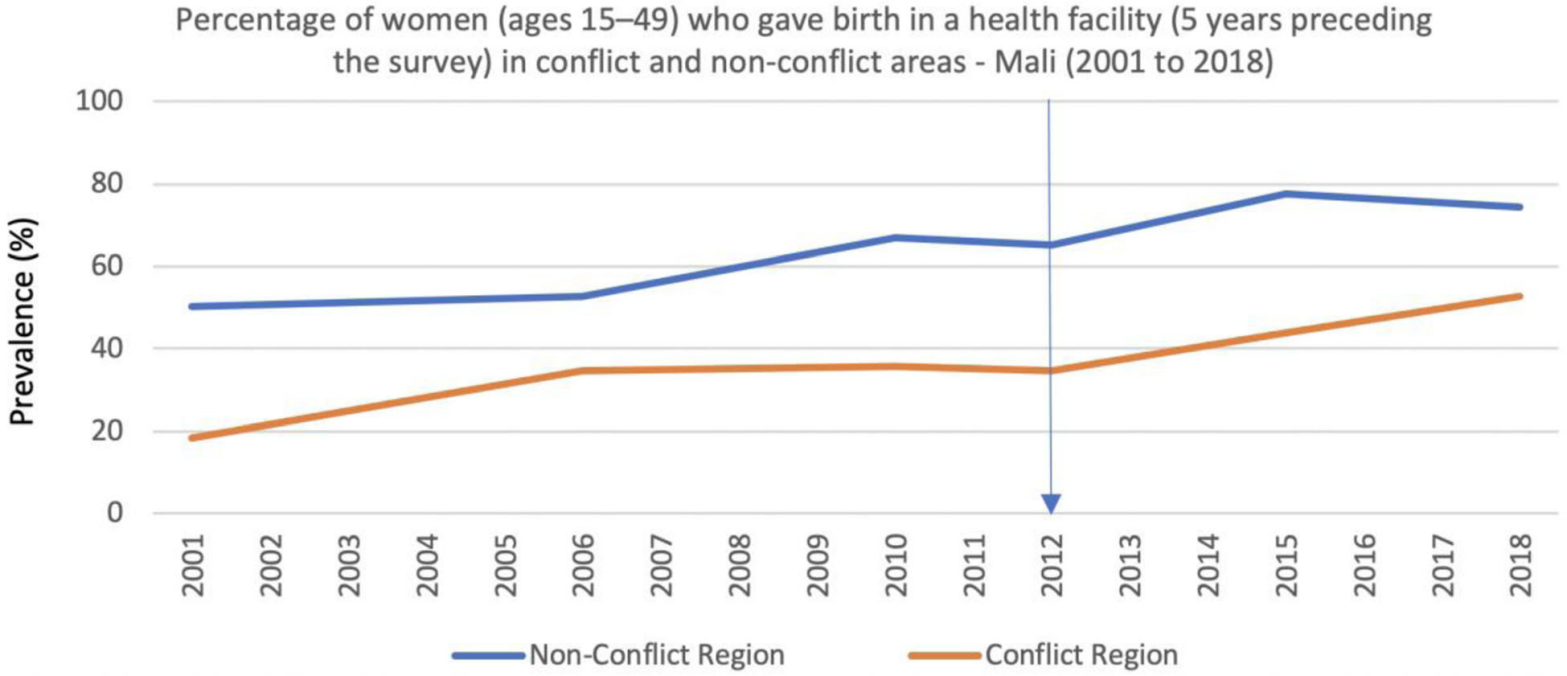
Percentage of women (ages 15–49) who gave birth in a health facility (5 years preceding the survey) in conflict and non-conflict areas - Mali (2001 to 2018)

Respondents believed that many women in Mali prefer to give birth at home and that sociocultural norms and societal pressures encourage this practice, despite the programmatic focus of many organizations towards increasing facility deliveries. The conflict-associated dangers that women faced when traveling to a health facility to deliver, respondents suggested, had made it even less likely that women would give birth in a facility. Nevertheless, coverage data show that facility deliveries increased in both conflict and non-conflict regions even after 2012, although the rates continue to be much lower in the conflict regions (Fig. 8).

**Fig. 9:**
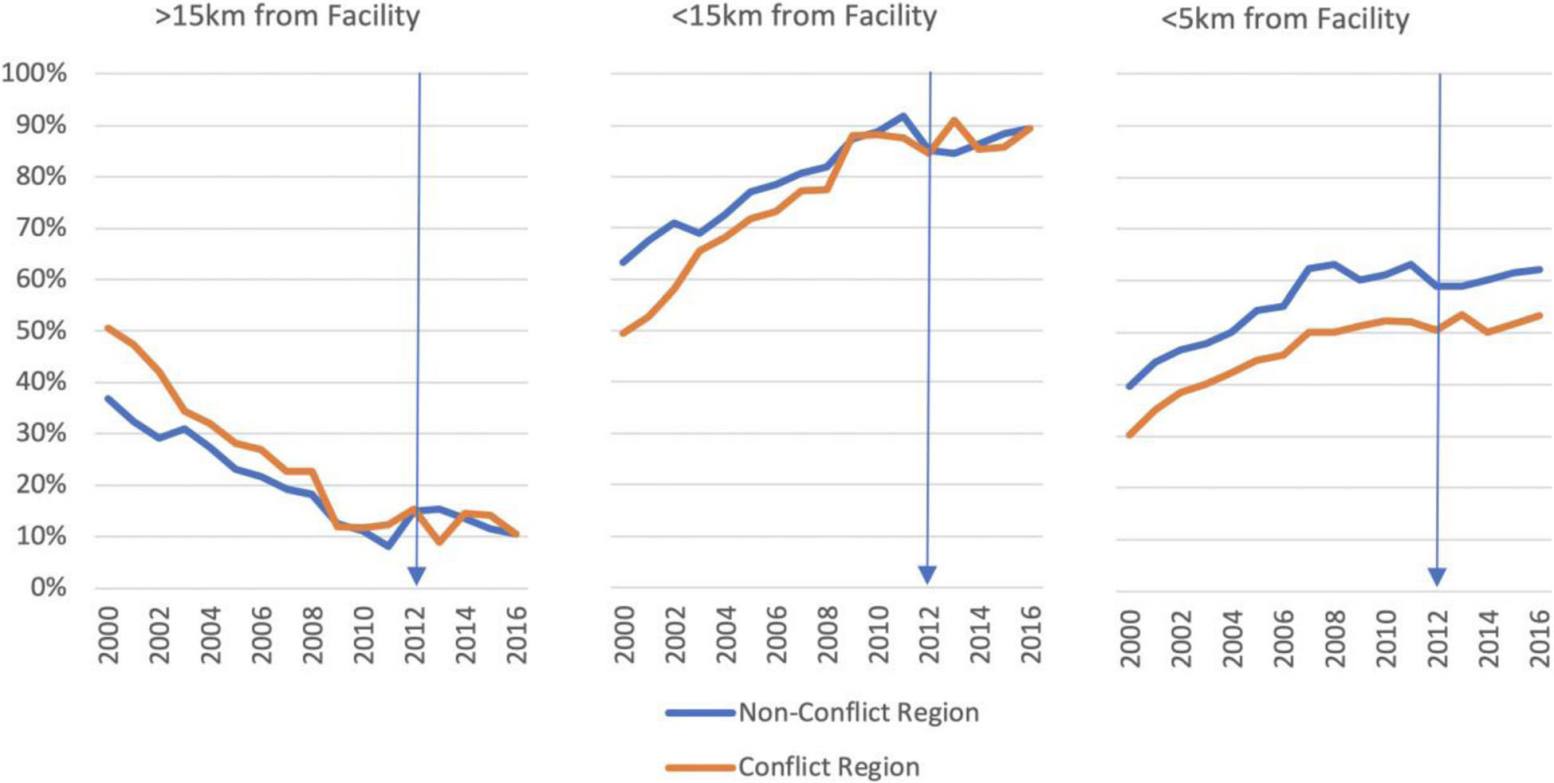
Proportion of the population by distance to the nearest health facility in conflict and non-conflict areas - Mali (2001 to 2016) Source: SLIS reports

Although the increase in facility deliveries in conflict regions may be initially surprising, it is an expected consequence of the high influx of migrant or displaced populations from rural and remote areas to larger cities. This pattern is evident in the conflict regions especially during the conflict’s most violent and insecure year, indicated by the number of internally displaced people in 2012 and 2013 (Fig. 1). Administrative data from the annual reports of Mali’s Système Local d’Information Sanitaire (SLIS) reveal that, starting in 2013, there was an increase in the proportion of the population living within 15 km and within 5 km of a health facility and an accompanying reduction in the proportion of the population living more than 15 km away, suggesting migration and/displacement as a likely explanation for these complementary trends (Fig. 9).

**Fig. 10:**
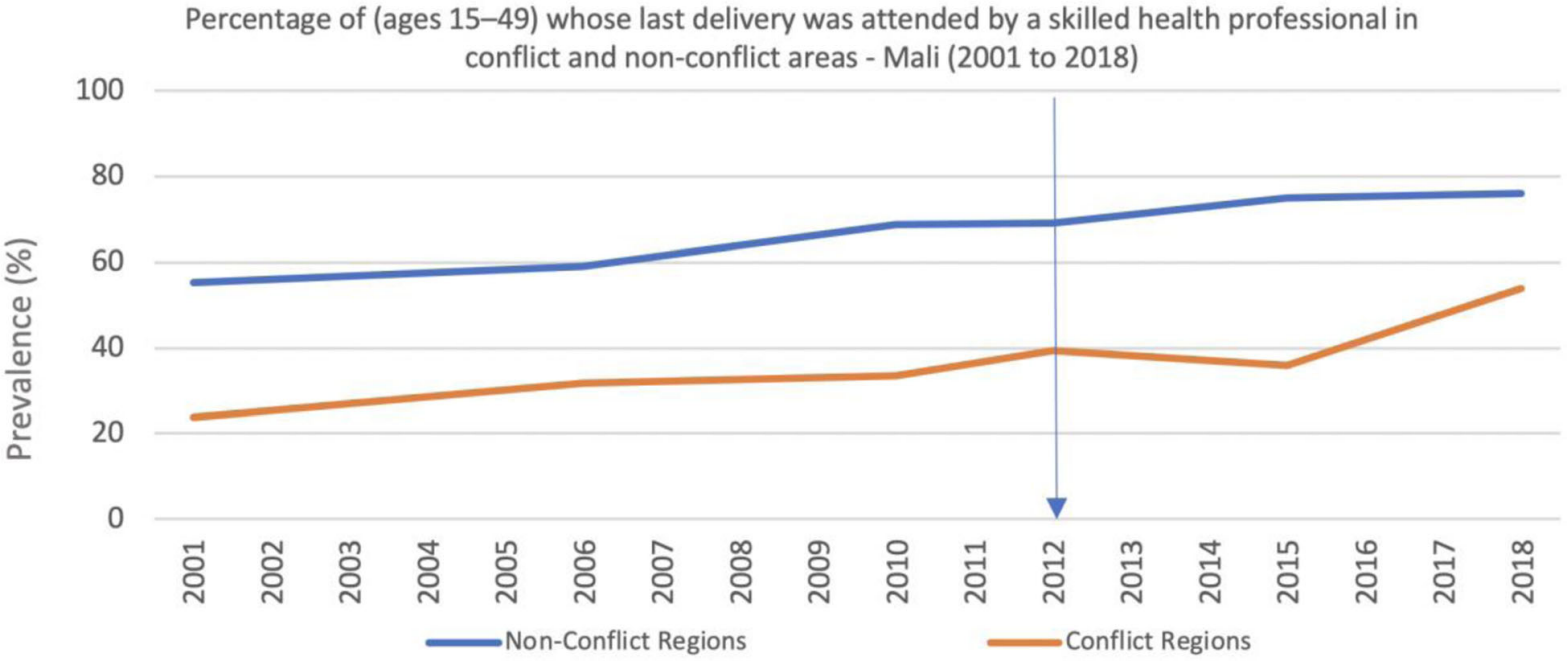
Percentage of (ages 15–49) whose last delivery was attended by a skilled health professional in conflict and non-conflict areas - Mali (2001 to 2018)

**Fig. 11:**
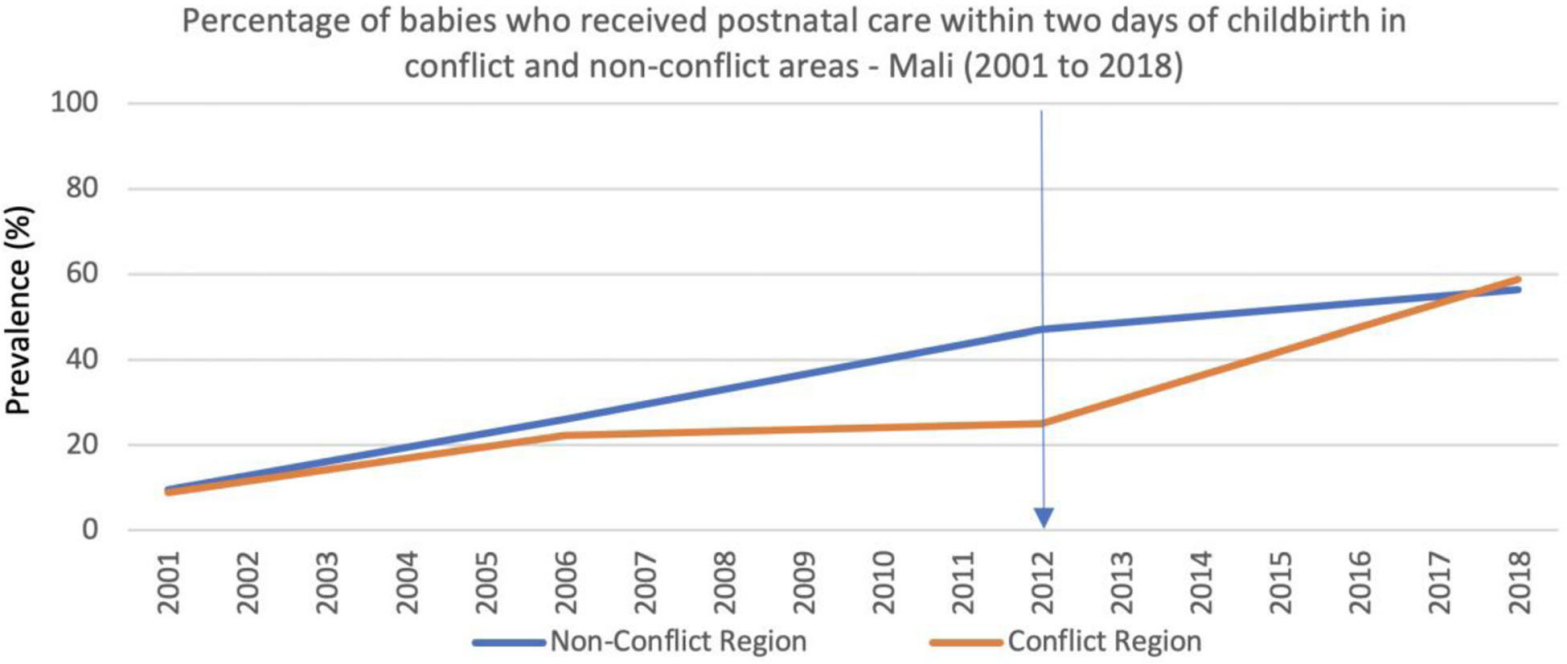
Percentage of babies who received postnatal care within 2 days of childbirth in conflict and non-conflict areas - Mali (2001 to 2018)

Deliveries attended by skilled health professionals nevertheless reduced between 2012 and 2015 in conflict regions, while continuing to modestly but steadily increase in the non-conflict areas (Fig. 10). Similarly, the percentage of newborns that received postnatal care within 2 days of delivery was not sustained in the conflict regions immediately after the conflict began, even though it continued to increase in the non-conflict areas. The most recent data from 2018 suggest no inequality between conflict and non-conflict regions (Fig. 11).

Personnel shortages were a persistent issue, especially for the government health facilities. This may explain the reduction in the prevalence of deliveries attended by skilled health professionals in the conflict areas between 2012 and 2015. Even before the conflict started, skilled birth attendance was lower in the northern regions when compared to the non-conflict regions, where coverage continued to increase from 2012 despite the conflict and political instability (Fig. 10). Since 2015, prevalence of skilled birth attendance has increased in the conflict regions, although still only reaching 54% of all deliveries by 2018 in comparison to almost 80% in the non-conflict regions (Fig. 10). This seems to coincide with two main factors; the government re-gained control of the major cities in the northern region and health workers were able to return, and people continued to migrate into areas with higher population density and closer to facilities (Fig. 9). Although several factors may explain the differences between conflict and non-conflict areas, the reversal in the positive trend in deliveries attended by skilled health professionals (Fig. 10) observed during the 3 years in the conflict areas immediately after the conflict started, contrasts with the continued increase in facility deliveries (Fig. 8), and corroborates the reports from the qualitative interviews about the difficulties in retaining health workers.

### Service delivery: creativity and courage

Organizations’ ability to deliver services in northern Mali was impacted by the unpredictable and rapidly changing nature of the conflict. The security situation in certain areas could change quickly and often respondents reported feeling vulnerable to attacks. People working in development and humanitarian organizations were frequently targets in kidnappings, and this impacted costs as well as recruitment and retention of health workers. Beyond increasing insurance costs (and ransom payments), many organizations chose to offer above-market remuneration for those willing to work in the conflict areas. The unintended consequence of this was movement of staff between organizations, leading to service disruption and forcing organizations to continuously recruit and train new staff.

Jihadist groups often made monetary demands to organizations in exchange for their safe passage and operations, and different organizations responded differently to these demands. Organizations that refused to pay often justified their decision by the uncertainty of any rebel-offered guarantee of safety and also by arguing that their limited budgets could only be used to directly support the delivery of services. Those organizations that agreed to such demands incurred operational cost increases and often had to re-adjust programming due to the budget impacts. The unpredictability and the volatility of the various actors involved in the conflict in northern Mali increased the difficulties in sustaining programs. Most organizations relied on local community member to deliver programs to the communities, but, as illustrated by one of our respondents, it was not unusual for a community member to enlist with rebel forces.

The [reality] is that you cannot plan an activity and be sure that it will happen. The other [reality] especially, in the Mali conflict, is that we are in a situation of insecurity where actors keep switching [sides] overnight. This necessarily impacts [program] implementation activities. You may leave a [village] today [confident] you have identified a gentleman who is pro-government and who will facilitate your work. … The next day you come back, and you may find [the gentleman] has left [to join] the armed groups that are against the government and that [now] the people who are pro-government are his enemies. In these areas, it's really complicated. (International NGO employee)

This level of unpredictability compromised program delivery and created a complex management problem, especially for international NGOs that depend on local support to implement activities. Periodic attacks on vehicles transporting medicines, supplies, and equipment were also frequent challenges, with transport vehicles frequently intercepted and the commodities and vehicles stolen.

I had an UNFPA ambulance stolen from me when I was bringing a patient with acute appendicitis to the care center. I had another ambulance that the State provided to me stolen as well, so you can see that the conditions we are working in are hard and people are working against us. (International NGO employee)

The reduced safety risk of inconspicuous transport prompted a shift towards the use of motorcycles to transport health workers and patients, but the government banned the use of motorcycles in certain areas of the country to limit the circulation of rebel groups, impacting service delivery. Organizations resorted to public transport to deliver services, impacting frequency of service provision and often forcing organizations to adjust service delivery to coincide with the day after *jours de foire*, local fair days when vendors from rural areas would travel to the cities to sell their goods. As the availability of public transportation to and from the cities increased around fair days, the coordination of service delivery and the *jours de foire* enabled organizations to expand the reach of their services. In contrast, some organizations ignored the motorcycle ban, risking their workers’ safety.

My field agents use the means at their disposal (cart, bike) to go from village to village, or if the village is 3 kilometers or 5 kilometers [away], they sometimes take the risk of using a shortcut on a motorcycle without being seen by security. (Local NGO employee)

Some organizations used army escorts to guarantee the safety of their operations, but this lead to complexities in service delivery schedules and reduced time windows. Other organizations preferred to avoid army protection as they believed that it could increase their risk of attacks. Due to the volatile nature of the conflict and of its actors, there was justification for both approaches.

If we are able to work in Mopti city today, it's because of the armed forces. If the armed forces weren’t present, or if the armed forces decide to leave, I do not know how confident you would feel. The presence of the security forces sometimes makes us feel secure, reassures us, but sometime is a source of concern. Especially since we know that radical groups only attack the armed forces. It's for these reasons that we avoid military combats. Moreover, if they are the targets and you are among them, you are more likely to be a target too. (International NGO employee)

The unpredictability of conflict also made access to communities inconsistent. In some cases, planned field visits to deliver equipment and resources had to be cancelled due to road closures, also preventing communities from accessing care. Many conflict areas were also affected by pervasive interethnic tension between the Dogon and Fulani. This posed a further threat to the communities and organizations working in those areas, with respondents perceiving the need to delicately navigate these dynamics, ensuring that they were not perceived as partial to any specific ethnic group.

You have to be careful who you talk to because people will think you are choosing sides and are helping the Dogons or the Fulanis. Even though you aren't picking sides they will assume you are and then you are subjecting yourself to an attack even though you are only trying to provide unbiased health services. (International NGO employee)

Interethnic tension was reported to be the strongest in Koro and Bankass towns in Mopti region, with access to care being particularly limited in these areas.

Sometimes when the [primary health facility] is in the Dogon area, the Fulani cannot get treatment. You cannot take care of them. Your life is threatened. And vice versa, there are agents who have been threatened because they took the Fulani records. Under threats, they were forced to leave. (Government employee)

### Coordination: the key to improving efficiency

As mentioned earlier, collaboration between different types of humanitarian actors was vital to ensuring service delivery. Organizations use the term *faire faire* when referring to the practice of subcontracting local organizations or community members to deliver services when there are security concerns, but also as a mean to gain local contextual insights. *Faire faire* also describes the ways by which organizations fund local health centres or conduct capacity building activities with local health staff.

We have areas where there is more representation, we also have areas where the CSCOMs are not functional, but that does not mean that we cannot intervene there. We set up a ‘faire faire’ system, meaning that, in an area, if we have someone who can do the work, the [organization] is invited to the [field office] or to the regional level, is [authorized] to carry out activities on the area. (Local NGO employee)

Our data suggest that the initial humanitarian response activities were poorly coordinated, with organizational isolation and fragmentation of activities often resulting in the duplication of services. Despite improvements in coordination as the conflict evolved, collaboration between humanitarian actors was still perceived to be insufficient and government representatives expressed dissatisfaction with the role that the government played in the delivery of humanitarian activities, arguing that their efforts were not visible.

There is co-ordination between NGOs but there is a huge gap in this area. And at the moment we find ourselves in this situation which is not new and very usual and in which the State does not play its role in coordinating. Since the roles are no longer well defined, the International NGOs even intervene in the place of the State. Tshese are aggravating factors. I will try not to give the names of NGOs; these are NGOs that finance everything. And as a result, the State has nothing to do with it and people see the NGOs, they do not see the State. It does not allow for the State to find its place, it creates frustrations at the level of the State. In the current conflict in Mali, the main problem is the security problem, this is not my domain. It's the army, there is the Barkhane Program, there is MINUSMA, there is the Malian Army. This is their domain but I remain convinced that the solution to the problem will be easier to attain if the State is present and the people will look after the State. Right now, I think NGOs are much more visible than the State. (Government employee)

Cluster coordination meetings were valued as a mean for organizations to stay up to date about the activities being delivered by other organizations in the region and also as a source of information on emerging funding opportunities. No respondent was explicitly critical about the meetings, or about the cluster approach, but some did note that they preferred not to attend the meetings, suggesting that their perceived added value is low. The study team’s own observations during a health cluster meeting in Bamako in 2018 confirmed the utility of these meetings for sharing information on the activities of different organizations and also as a forum to discuss needs and funding opportunities, but not as a forum for coordination of action.

Ultimately, fears related to the future of Mali were palpable during interviews. Several respondents from local NGOs were concerned that the conflict and instability in Mali would result in international actors leaving. They reported that organizations were becoming increasingly resistant to sending foreign health workers to Mali, and these fears were echoes by concerns from government representatives about the sustainability of the services provided by external humanitarian actors.

## Discussion

Our findings confirm what has been previously described in other settings regarding the complexity of delivering maternal and child health and nutrition services in conflict-affected areas. Different from populations who become entrapped in conflict areas, the population of northern Mali has been mostly displaced, many fleeing their homes for cities both within the region and also to non-conflict regions. Some previous analysis suggests that the majority of the displaced population has fled to the southern regions and more specifically to the capital city of Bamako, but there is some indication that rural-urban migration within the conflict-affected regions was the norm among the most vulnerable groups [30]. Access to care is a key issue of concern for internally displaced persons (IDPs) [1], which were estimated to include 47,000 individuals within Mali in late 2018 [6], but have increased to almost 200,000 people according to the most recent estimates from OCHA [31, 32].

It is also possible, and likely, that the conflict has affected health system functionality and service delivery even outside of the insecure areas, but the limited data available make it difficult to confirm this hypothesis. Some of our data suggest non-conflict areas may have benefited from an influx of funding for vaccines into the country, while data on antenatal care and postnatal care in non-conflict areas suggest a deceleration of the pre-conflict progress. However, the extent to which such trends are a function of the conflict is unknown.

The wide range of international humanitarian actors that is often observed in other conflict settings has in fact been more limited in Mali, as the unpredictability of the conflict and the changing alliances of different interest groups in the region since 2012 have constrained the operations of large international humanitarian organizations that have had to rely on smaller local organizations to deliver their programs in Mali. While many of these large organizations would have delivered health services directly in non-conflict situations, in the context of the Mali conflict many have effectively become fund managers with little control over the delivery of the programs that they designed and were funded to deliver. The smaller, local organizations are essential as they are often the only ones able to operate in highly volatile and unsafe areas and the only ones who can access the communities and assess their needs [33]. But such arrangement also carries the weight of increased operational costs, not only due to the security costs associated with program delivery in these unsafe areas, but also because this sub-contracting structure adds an administrative layer to the program management that would not exist otherwise.

Moreover, concerns have been raised about the disconnect between the very high administrative capacity of large NGOs that subcontract the work, and the disproportionate security and other risks borne by local NGOs that are actually delivering the programs in Mali [34]. In addition, in their implementing roles, local NGOs consequently have very limited access to non-operational funds, reducing their capacity to function outside of the current arrangements [33].

Complicating the coordination of the humanitarian health response in Mali is the fact that the government was generally distanced or even excluded from programming discussions and decisions due to varying levels of uncertainty about the government’s role in the conflict and the optics of working with government within certain populations in the conflict areas. The situation continues to evolve and perception of roles is constantly changing, as it has been throughout the conflict.

It has been stressed that affected communities should be at the core of the humanitarian system and also central to the decision-making process [35], but this principle was not mentioned at all during any of the interviews we conducted. References were made to changing needs of the population, and how that was difficult to address within funding structures, but not to the role of affected persons in decision-making processes.

Access to services for individuals in Northern Mali is limited and organizations report that women attempting to access maternal and child health care in Northern Mali are concerned since there are reports that Jihadists monitor health visits to ensure women don’t access family planning services [2]. Estimates from the most recent humanitarian response plan state that, at present, 1.7 million individuals have compromised access to health care [6].

Finding solutions to the lack of flexibility and to simplify and increase responsiveness of the current humanitarian health funding structures in Mali have massive positive impacts on the ongoing and future response by allowing for a better alignment of activities with the immediate needs emerging in this very dynamic conflict setting.

Logistical constraints of our study, including timelines and security concerns, necessitated the use of multiple teams of data collectors working simultaneously. How the data collectors were perceived by the respondents may have influenced the nature of the information that respondents disclosed. The data collectors were also not directly involved in the data analysis process. Debriefs between the data collectors and the project coordinator help ensured that subsequent interviews responded to the preliminary findings that emerged, and that the interpretation of the interviews was accurate. The iterative nature of our qualitative data collection process required several rounds of training to ensure that our data collectors were obtaining the most relevant information to meet our study objectives. Even with ongoing training, interview quality was variable and we ultimately determined that only 25 of the 50 conducted interviews were of sufficient quality to contribute to our analysis and findings. Lastly, as with any qualitative study, the generalizability of our qualitative findings is limited, and the findings from our interviews focusing on Mopti may not reflect experiences in other geographies in Mali.

## Conclusion

Our findings suggest that the constraints of the prevailing humanitarian health funding mechanisms in Mali have been a major barrier to the timely delivery of essential health services to populations in need in Mopti regions, that and the nature of the conflict is likely a key modifier of how such mechanisms operate. Identifying the parameters that can minimize perceived investment risks and increase efficiency in financial flows into conflict zones is crucial to ensuring that the response of humanitarian organizations is effective. Coordination strategies that include international donors, local government and humanitarian organizations must be improved and would benefit from more structured guidelines and stronger centralization. The current coordination strategies seem to have resulted, at least in Mali, in a vicious cycle that undermines authority and suggests irrelevance. As the conflict in Mali lingers, there continues to be an urgent need to improve service delivery to conflict-affected populations. Centralized and organized coordination across sectors and humanitarian actors has the potential to promote accelerated response to emerging issues, more efficient use of financial and human resources and more appropriate prioritization of programs.
